# Differences in drug removal between standard high-flux and medium cut-off dialyzers in a case of severe vancomycin toxicity

**DOI:** 10.1093/ckj/sfae063

**Published:** 2024-03-13

**Authors:** Simon Aberger, Michael Kolland, Kathrin Eller, Alexander R Rosenkranz, Alexander H Kirsch

**Affiliations:** Division of Nephrology, Department of Internal Medicine, Medical University of Graz, Graz, Austria; Department of Internal Medicine I, Paracelsus Medical University, Salzburg, Austria; Division of Nephrology, Department of Internal Medicine, Medical University of Graz, Graz, Austria; Division of Nephrology, Department of Internal Medicine, Medical University of Graz, Graz, Austria; Division of Nephrology, Department of Internal Medicine, Medical University of Graz, Graz, Austria; Division of Nephrology, Department of Internal Medicine, Medical University of Graz, Graz, Austria

**Keywords:** chronic hemodialysis, clearance, dialysis, dialysis dose, haemodialysis

## Abstract

Vancomycin is a widely used glycopeptide antibiotic with the need for therapeutic drug monitoring to avoid renal toxicity. We report a case of severe vancomycin-associated anuric acute kidney injury managed with successful drug-removal by hemodialysis (HD) using different types of dialyzers. Medium cut-off (MCO) and high-flux dialyzers were effective in drug removal. Higher vancomycin elimination rate and lower plasma half-life were achieved with MCO dialyzer despite low-flow vascular access and intolerance to ultrafiltration. MCO dialyzers may be reasonable for drug removal in patients with intolerance of ultrafiltration, low-flow vascular access or impracticality of hemodiafiltration. Future studies should explore the use of MCO dialyzers in comparison with high-flux HD and hemodiafiltration in both the acute and chronic setting.

## INTRODUCTION

Vancomycin is a glycopeptide antibiotic frequently used to treat Gram-positive bacterial infections. Vancomycin has a molecular weight of 1500 Da with a molecular radius of 2.5–3 nm (Einstein–Stokes radius potentially larger) [1], and its elimination is largely dependent on renal clearance. Therapeutic drug monitoring of plasma trough levels (target range for adults 15–20 mg/L) is necessary due to its dose-dependent toxicity. Impaired renal clearance can cause vancomycin accumulation and prompt the necessity for hemodialysis (HD) in cases of severe nephrotoxicity. Vancomycin is known to be eliminated by HD with elimination rates differing between conventional high-flux (HF) and low-flux (LF) dialyzers [2]. Since vancomycin is commonly used in HD patients, prior studies mainly focused on guiding vancomycin dosing in patients receiving intermittent HD [3]. The handling of middle-sized molecules by medium cut-off (MCO) dialyzers and HF dialyzers has been the topic of recent studies suggesting superior clearance of middle-molecules by MCO dialyzers similar to hemodiafiltration [4]. However, insufficient clinical data exist concerning drug removal in patients with acute medical conditions.

Translating the theoretical efficiency of dialyzers into “real-life” clinical settings is hampered by individual patient conditions including blood flow and ultrafiltration tolerance, as well as multiple-compartment pharmacokinetics [5]. Hence, pharmacokinetic data from clinical scenarios are needed to guide treatment decisions. Therefore, we wish to report a case of severe vancomycin toxicity treated with HD using different types of dialyzers.

## CASE REPORT

**Figure 1:**
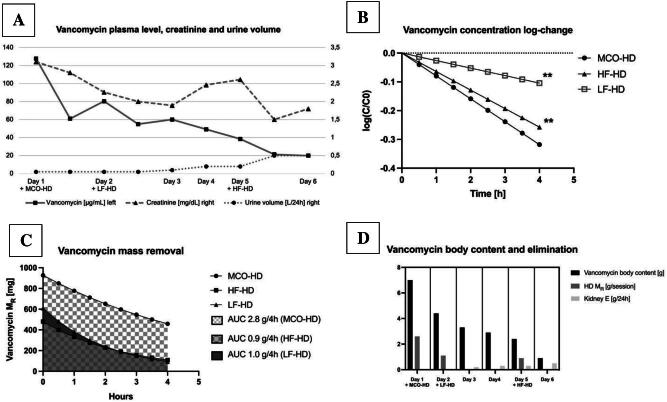
(**A**) Trend of vancomycin plasma level, kidney function and 24-h urinary output via Foley catheter. Three dialysis sessions were performed to reduced vancomycin plasma level: MCO-HD on Day 1 (−53%), LF-HD on Day 2 (−30%) and HF-HD on Day 5 (−44%). (**B**) Log-change transformation was used to transform exponential elimination curves (calculated with first order pharmacokinetics) of all three dialysis sessions for slope comparison by a linear regression model between MCO-HD:LF-HD and MCO-HD:HF-HD; ** indicates *P* < .01 compared with MCO-HD. (**C**) Effluent vancomycin was calculated as area under the curve (AUC) from exponential elimination function calculated by two measurements of dialysate effluent vancomycin concentration using first-order kinetics in 4-h sessions of MCO-HD, LF-HD and HF-HD with a constant dialysate flow of 500 mL/min. (**D**) Vancomycin mass removal (M_R_) by HD compared with pre-dialysis vancomycin body content are shown for each day as a bar graph; native kidney excretion (Kidney E) was assumed as the remaining difference compared with pre-dialysis vancomycin body content.

A 56-year-old woman was treated with a cumulative dose of 27 g of vancomycin for bacterial infection over 1 week. Plasma trough level was 18 mg/L after the first 1.5 g of vancomycin. Blood tests taken 3 days later were reported invalid due to analytical error. No dosing adaptation was made between measurements. After 7 days of vancomycin, a plasma trough level of 128 mg/L was measured and the patient presented with anuric acute kidney injury [AKI stage 3; creatinine 3.1 mg/dL (272.8 µmol/L), baseline 0.58 mg/dL (51.0 µmol/L)]. Concomitant medication included hydromorphone, long-term use pantoprazole, amlodipine and prophylactic low molecular weight heparin. No nephrotoxic medication or recent contrast media were used, ultrasound did not show hydronephrosis and no signs of sepsis were present (C-reactive protein 10 mg/L, negative blood cultures). Vancomycin was halted and three intermittent HD sessions were conducted via a central venous catheter, according to the clinical indication to actively remove vancomycin until plasma level returned to <20 mg/L (Fig. 1). HD sessions were done using MCO (Theranova 400, Baxter, Deerfield, IL, USA), HF (Revaclear 400, Baxter) or low-flux (Polyflux 17 L, FMC, Bad Homburg v.d. Höhe, Germany) dialyzer. The latter was used due to logistical difficulties. Vancomycin plasma trough level decreased <20 mg/L after the third HD session and 24-h urine output started to increase, reaching 1200 mL, with serum creatinine stabilizing around 1.5 mg/dL (130 µmol/L) over the following 2 weeks without the need for further HD (Fig. 1). No clinical signs of hearing loss were reported; audiometry was not conducted in this case.

To determine the efficiency of drug-removal with different dialyzers, vancomycin plasma levels were measured before and after each 4-h HD session to calculate drug half-life, elimination coefficient and clearance assuming first-order kinetics [6]. The log-change transformation of vancomycin plasma level elimination curve was further calculated to fit a linear regression model for slope comparison between LF, HF and MCO dialyzers. To account for compartment redistribution, vancomycin concentration was also repeatedly measured in the dialysate effluent to estimate vancomycin mass removal (M_R_) more precisely by the area under the curve method, given a constant dialysate flow of 500 mL/min. Vancomycin total body content was estimated by calculating the volume of distribution using population-based pharmacokinetic modelling, Vd = 0.78 L/kg (71.5 kg, body mass index 25 kg/m^2^, Vd = 55 L), as suggested by Buelga *et al*. [7]. Calculations were done with Excel 2023 (Microsoft, USA) and Prism 10 (GraphPad, USA), and statistical significance was accepted for *P* < .05.

## DISCUSSION

Vancomycin-associated kidney injury may result from dose-dependent non-crystalline intratubular obstruction, direct tubulotoxicity via radical oxygen species or potentially, dose-independent interstitial nephritis [8]. Clinical suspicion for vancomycin-associated and dose-dependent AKI is high in our case, considering the severely elevated plasma trough level, lack of alternative cause of AKI and prompt improvement of kidney function after rapid reduction of vancomycin plasma level. However, kidney function was restored only partially in the short term, suggesting residual damage. This highlights the need to closely follow guidelines for therapeutic drug monitoring of vancomycin; measurement of serum creatinine and plasma trough level should be performed vigilantly in all patients with administration longer than 3–5 days; performance of audiometry may be considered to detect subclinical hearing loss when vancomycin toxicity is suspected [6].

MCO-HD reduced vancomycin plasma level by 53% despite low-flow vascular access and intolerance of ultrafiltration during the first treatment session. Consecutive HF-HD and LF-HD sessions decreased vancomycin plasma level by 44% and 30%, respectively (Fig. 1). Vancomycin concentration change was significantly higher for the MCO dialyzer compared with the LF and HF dialyzers (R^2 ^= 1, F = 1438, *P* < .01; Fig. 1). Calculated vancomycin clearance was highest for the MCO dialyzer (170 mL/min) compared with the HF (135 mL/min) and LF (98 mL/min; Table 1) dialyzers. Vancomycin plasma levels rebounded between consecutive HD sessions by up to 30%, representing compartmental redistribution as described in a previous report [9]. To account for these confounders, cumulative vancomycin mass removal in the dialysate effluent was compared with steady-state vancomycin total body content, which showed a 40.7% extraction for HF dialyzer with higher blood flow and additional ultrafiltration compared with 40% extraction with MCO dialyzer (Fig. 1, Table 1).

**Table 1:** Data comparison from individual 4-h HD sessions using different dialyzers.

Dialyzer	MCO	HF	LF
Modality	MCO-HD	HF-HD	LF-HD
Blood flow (mL/min)	200	300	250
Ultrafiltration (L/session)	0	2	0.3
Session duration (min)	239	240	237
Reduction ratio (%)	53	44	30
Vancomycin half-time (min)	230	280	342
Vancomycin CL (mL/min)	170	135	98
Vancomycin body content (g)	7	2.2	4.4
Vancomycin effluent (g/4 h)	2.8	0.9	1.0
Effluent/body content (%)	40	40.7	22.7

**Figure 2:**
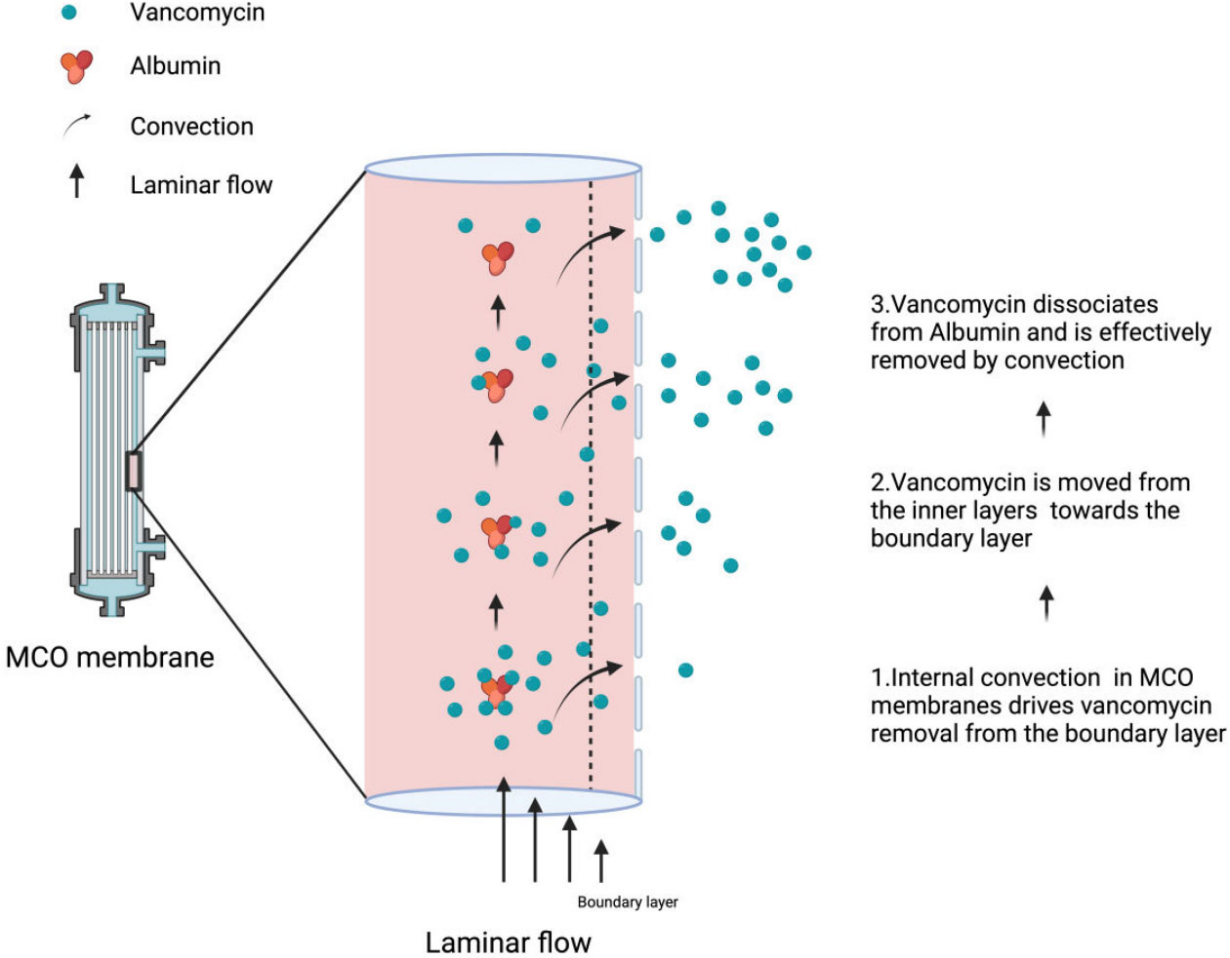
Schematic illustration of vancomycin mass removal through internal convection in a single hollow fiber filtration segment of an MCO dialyzer. Publication license was obtained from ©Biorender.

These findings are in line with a prior study showing a trend towards higher vancomycin clearance with MCO compared with HF dialyzers for intradialytic vancomycin administration in chronic HD patients [10]. Furthermore, treatment with continuous venovenous hemodiafiltration led to a similar reduction in vancomycin plasma level in a comparable case report on vancomycin toxicity in the intensive care setting [11]. Due to the technical design of MCO dialyzers allowing improved internal convective transport of middle-sized molecules through enhanced back-filtration and adapted pore size as well as pore density, it is considered a cost-effective alternative to hemodiafiltration (as reviewed in [12]). Since vancomycin is a smaller middle-weight molecule (1500 Da) with ≈50% bound to albumin, mass transfer will largely depend on convective solvent drag from the inner towards the boundary layer of laminar blood flow to facilitate its dissociation from albumin [13]. This effect may explain superior vancomycin mass transfer by MCO membranes in our case, despite the similarly high sieving coefficients >0.9 for both HF and MCO membranes [14] (Fig. 2). Although generalization of these findings in consecutive HD sessions of a single patient has to be approached with caution, pharmacokinetic data from this rare clinical scenario may guide individual treatment decisions. Furthermore, optimization of middle-molecule clearance is currently gaining attention due to recent results published by Blankestijn *et al*. in the CONVINCE trial [15]. Exploring the use of MCO dialyzers in comparison with HF-HD and hemodiafiltration in both the acute and chronic setting may be worthwhile in future studies.

In conclusion, MCO and HF dialyzers are effective in drug removal in vancomycin-induced AKI with anuria. We observed a trend towards lower vancomycin half-life and higher plasma level reduction rate in a single session of MCO-HD compared with HF-HD and LF-HD. MCO dialyzers may be reasonable for drug removal in patients with central venous dialysis catheter, clinical conditions affecting vascular access function, ultrafiltration intolerance or impracticality of hemodiafiltration.

## Data Availability

The data underlying this article are available in the article and in its online supplementary data.
